# Age at Onset and Social Cognitive Impairment in Clinically Stabilized Patients with Schizophrenia: An Ecological Cross-Sectional Study

**Published:** 2018-04

**Authors:** Alice Caldiroli, Marta Serati, Giulia Orsenigo, Elisabetta Caletti, Massimiliano Buoli

**Affiliations:** Department of Psychiatry, University of Milan, Fondazione IRCCS Ca’Granda Ospedale Maggiore Policlinico, Milan, Italy.

**Keywords:** *Age at Onset*, *Cannabis Abuse*, *Gender*, *Schizophrenia*, *Social Cognition*

## Abstract

**Objective:** Purposes of the present study were to assess the social cognitive impairment in schizophrenia and to detect if some clinical variables (particularly age at onset) are predictive of general/social cognitive deficit in schizophrenia patients.

**Method:** Thirty-five clinically stabilized schizophrenia outpatients were assessed by the Brief Assessment of Cognition in Schizophrenia (BACS) and by Torralva’s social cognition battery. Binary logistic models were performed to find an eventual association between continuous clinical variables and cognitive test failures. The total sample was divided in groups according to dichotomous variables (gender, diagnostic subtypes and type of abuse) and the presence of cognitive deficits was compared between groups by χ2 tests.

**Results:** An earlier age at onset was found to be predictive of frontal cognitive impairment (Tower of London p=0.038, OR=0.702). Female gender was more probably associated with mistakes at MET-HV (χ2= 4.80, p=0.05, phi=0.40) and HOTEL tests (χ2= 5.25, p=0.04, phi=0.4) than male one. Cannabis abusers showed more frequently deficits on verbal fluency (χ2= 9.35, p=0.04, phi=0.52) and executive functioning (Tower of London) (χ2= 11.67, p=0.02, phi=0.58) than alcohol/cocaine ones.

**Conclusion:** Female patients with an early age at onset and cannabis abuse seem to have the worst general and social cognitive profile among patients suffering from schizophrenia.

Several studies have shown that global cognitive impairment and deficits in the processing of emotions are typical features of schizophrenia (1-6). Schizophrenia patients appear to have deficits in different neurocognitive domains and, among them, memory, attention, and executive functions would be the most compromised (7, 8). In addition, schizophrenia patients often experience low level of performances and a reduced ability to live independently despite the remission of the acute symptomatology (9-11), with a negative impact on the social and occupational functioning and poor quality of life (10, 12). 

Of note, social cognition, defined as the mental operations underlying social interactions (10), has been recently interpreted as a mediator between neurocognition and functional outcome in schizophrenia (13). Furthermore, in light of the importance of cognitive impairment in affecting schizophrenia outcome, the interest in cognitive functioning has raised in the last twenty years (14,15) and cognitive impairment has been considered as an important pharmacological target for the development of new drugs in schizophrenia, having the available molecules no or small effect on cognition (16).

With regard to clinical symptoms, the available data show that cognitive impairment seems to be more strongly associated with the severity of clinical negative symptoms respect to positive ones (17-19). In addition, higher estimated premorbid IQ, years of education and predominant disorganized symptoms seem to be important predictors of poor neurocognitive performances (20-22). 

In schizophrenia patients, gender differences have been found in verbal learning and recall: a poorer neurocognitive performance, particularly in verbal memory, has been associated with male gender (20). Furthermore, altered neurocognitive functioning in several domains has been reported in schizophrenia patients with lifetime cannabis abuse (3, 23 and 24). In contrast, a meta-analysis found superior neurocognitive performances in cannabis-using patients compared to non-using ones (25). Moreover, a further study did not find significant differences on neurocognitive functioning or number of lost days of work between moderate/severe drug users and mild users or abstainers (26). Finally, alcohol abuse in schizophrenia is associated with more impaired functioning across many domains, including memory (27). It is currently debated if diagnostic subtypes are associated with severity of cognitive impairment. In two studies paranoid patients resulted to have better Verbal IQ, executive functioning and memory compared to undifferentiated ones (28-30), while another study showed that paranoid patients did not have higher intellectual functioning than those with a non-paranoid sub-type (31). 

Duration of illness probably influences the severity and type of cognitive deficits: a study showed that chronic patients presented more severe cognitive impairment especially in verbal memory, visual memory and attention respect to recent-onset schizophrenia patients (22). Furthermore, a longer duration of illness was found to correlate with impairment on psychomotor processing speed, verbal fluency and verbal learning (32(.

Duration of Untreated Psychosis (DUP)/ Untreated Illness (DUI) has been largely studied that an important factor in influencing cognitive abilities of schizophrenia patients. Longer DUP has been associated with deficits in Digit Symbol and Comprehension subtests (20), and, in first-episode schizophrenia, with deficits in verbal IQ, verbal learning, and verbal working memory (33).

Some studies showed that early age at onset is associated with severe cognitive impairment in schizophrenia patients (34-36). A study found a relationship between early age at onset and poor performances on IQ, Digit Symbol Coding and Tower of London tests (37). These findings are in agreement with a study (38), which demonstrated that worse Tower of London Task scores may primarily characterize early-onset schizophrenia. In line with these results, late-onset schizophrenia could be associated with better socio-functional outcome and higher possibility to get married (39).

On the basis of the mentioned data, the objectives of the present study are: 1) to assess the social cognition in a sample of stabilized schizophrenic outpatients and 2) to find an eventual relation between clinical variables and general/social cognitive deficits in schizophrenic patients.

## Materials and Methods

Thirty-five outpatients (twenty-nine males and six females) recruited from community services afferent to the Department of Psychiatry (University of Milan), with a diagnosis of schizophrenia according to DSM-5 (40) and treated with an antipsychotic mono-therapy, were included into the study.

Structured Clinical Interview for DSM –SCID-5-CV– (40), Global Assessment of Functioning – GAF (41), The Calgary Depression Scale for Schizophrenia - CDSS (42) and Positive and Negative Syndrome Scale - PANSS (43) were administered to the patients: those who showed a re-exacerbation of the disorder, as defined by a PANSS score > 60 (44), were excluded. Other exclusion criteria were comorbidity with mental retardation or other neurological conditions involving Central Nervous System (e.g. cerebral tumors), presence of a comorbid Axis I disorder (except substance or alcohol misuse), comorbidity with medical diseases (e.g. hypothyroidism) or metabolic disorders causing psychiatric symptoms, pregnancy and breastfeeding.

The design of the study was naturalistic and cross-sectional. General cognitive functioning and social cognitive functioning was assessed by this way:

*General Cognitive assessment*

General cognitive assessment was obtained using the Brief Assessment of Cognition in Schizophrenia - BACS (45, 46) that is a neuropsychological battery which includes Verbal Fluency, Token Motor Task, Symbol Coding, Tower of London Test, Verbal Memory (list learning) and Working memory (digit sequencing).

Verbal Fluency: patients have to say that many words as possible from a category in a given time (in our case 60 seconds). This category can be semantic such as furniture or fruits, or phonemic, such as words that begin with letter b.

Token Motor Task: patients are given 100 plastic tokens and asked to place them into a container as quickly as possible for 60 seconds.

Symbol Coding: it consists of nine digit-symbol pairs followed by a list of digits. Under each digit the patient should write down the corresponding symbol as fast as possible.

Tower of London: it consists of two boards with pegs and several beads with different colors. The rater uses the board in a variety of ways to test problem solving skills.

Verbal Memory (list learning): patients are presented with 15 words and then asked to recall as many as possible.

Working Memory (digit sequencing): patients are presented with clusters of numbers of increasing length.

*Social cognition battery*

In light of an overlapped cognitive impairment between schizophrenia and fronto-temporal dementia (47), social cognition was assessed using a 5-test battery sensitive in detecting executive and social cognitive impairment in early stages of the behavioral variant of Frontotemporal dementia (48). 

1. Multiple Errands Test for Use in Hospital Settings (MET-HV): the test requires subjects to carry out a number of tasks simulating “real life” situations where minor inconveniences can take place (49).

2. The Hotel Task: the task comprises six activities that would plausibly need to be completed in the course of running a hotel (50). 

3. Iowa gambling task: this test mimics real-life personal decision-making activities that include reward and punishment (51).

4. Reading the Mind in the Eyes: participants are required to choose between four options (adjectives) that best describes what the individual in the presented photo are thinking or feeling (52, 53).

5. Faux Pas Test: participants have to find something inappropriate in tales that they have to read and that may contain a social “faux pas” (a violation of accepted social norms) (54). 

*Statistical analysis*

Descriptive statistics were performed in order to evaluate general and social cognition in the total sample. Binary logistic models were then performed. In these analyses, the failure in a single test was considered that the dependent variable, while age, age at onset, duration of illness, DUI, Calgary scores, GAF scores and PANSS scores were the covariates. Χ2 tests were used to compare dichotomous variables (gender, diagnostic subtypes, type of abuse) and cognitive test failures. SPSS for Windows (version 22.0) was used as statistical program.

## Results

Descriptive statistics of the total sample are reported in table 1. In our sample patients showed impairment in different several domains. The worst performances resulted in Faux Pas Test (failure in 90.3% of patients), Token Motor Task (failure in 82.9% of patients) and Symbol Coding (failure in 80.0% of patients).

In table 2 the mean test scores are reported together with the percentage of failing patients.

The goodness-of-fit test results (Hosmer and Lemeshow Test: χ2=8.83, df =7, p=0.265) showed that the model including continuous variables/scale scores as possible predictors of Tower of London failure was adequate, allowing for a correct classification of 77.1% of the cases.

**Table1:** Demographic and Clinical Variables of the Clinically Stabilized Patients with Schizophrenia

**Variables**		**N=35**
Gender	Male	29 (82.9)
	Female	6 (17.1)
Age		41.40 + 10.22
Age at onset		20.94 + 3.70
Prevalent Symptoms	Psychotic	11 (31.4)
	Negative	15 (42.9)
	Disorganization	9 (25.7)
Abuse before the onset	No	13 (37.1)
	Yes	22 (62.9)
Duration of illness		20.46 + 11.29
Type of abuse	None	13 (37.1)
	Alcohol	5 (14.3)
	Cannabis	11 (31.4)
	Cocaine	4 (11.4)
	Heroin	2 (5.7)
Duration of untreated illness		2.80 + 4.78
Treatment	Quetiapine	3 (8.6)
	Olanzapine	5 (14.3)
	Risperidone	8 (22.9)
	Aripiprazole	5 (14.3)
	Zuclopenthixol	7 (20.0)
	Haloperidol	5 (14.3)
	Paliperidone	2 (5.7)
PANSS total score		48.06 + 3.92
CDSS		4.74 + 3.88
GAF		43.34 + 11.79

Note: Standard deviations for continuous variables and percentages for dichotomous ones are reported into brackets

PANSS: Positive and Negative Syndrome Scale

CDSS: The Calgary Depression Scale for Schizophrenia

GAF: Global Assessment of Functioning

**Table2:** Summary of the Results in Cognitive Domains in Clinically Stabilized Patients with Schizophrenia

COGNITIVE TESTS Sample N=35	Minimum Score	Maximum Score	Mean Score	Standard Deviation	% of Failure
BACS					
Verbal Memory vn > 33.01	21	57	35.69	± 8.477	60.0
Working Memory vn > 14.93	3.25	26.25	16.87	± 5.339	51.4
Token Task vn > 68.77	25	90	61.15	± 16.126	82.9
Symbol Coding Task vn > 40.49	15	63	38.56	± 10.858	80.0
Verbal Fluency vn > 31.68	16.3	58.5	34.04	± 9.587	68.6
Tower of London vn > 12.37	0	20	12.79	± 4.010	68.6
SOCIAL COGNITION					
MET					
Tasks attempted	4	12	10.07	± 2.288	56.7
Task failures	0	8	1.93	± 2.288	56.7
Inefficiencies	0	3	1.47	± 0.776	56.7
Rule breaks	0	2	0.63	± 0.615	0.0
Interpretation failure	0	4	0.63	± 0.999	40.0
Total failures	0	11	4.67	± 3.294	30.0
HOTEL					
Tasks attempted	0	5	3.17	± 1.315	53.3
Tasks correct	0	5	3.17	± 1.315	53.3
Time deviation (sec)	0	720	277.93	± 209.415	24.1
Button pressing	0	2	1.3	± 0.915	40.0
Opening deviation	0	4	2.67	± 1,826	
Closing deviation	0	4	2.17	± 1,913	
Garage time deviation	0	8	4.83	± 3.534	50.0
IOWA GAMBLING TASK	-42	52	-1.1	± 21.806	58.1
THEORY OF MIND TESTS					
THE EYES TEST	10	25	19.68	± 4.482	35.5
FAUX PAS TEST	0	20	12.52	± 3.846	90.3

BACS: Brief Assessment of Cognition in Schizophrenia

HOTEL: The Hotel Task

MET: Multiple Errands Test

**Table3:** Summary of the Statistics for the Best-Fit Logistic Regression Model Applied

**Clinical Variables/ Scale Scores**	**B**	**S.E.**	**Wald**	**df**	**p**	**Exp (B)**
Age	0.013	0.055	0.058	1	0.809	-1.013
Age at onset	-3.54	0.170	4.321	1	0.038	0.702
DUI	0.056	0.117	0.230	1	0.632	1.058
CDSS scores	-0.236	0.143	2.718	1	0.099	0.790
GAF scores	0.037	0.045	0.658	1	0.417	1.037
PANSS scores	0.285	0.187	2.331	1	0.127	1.33

In this analysis the dependent variable was the Tower of London success

B=coefficient; S.E.= standard error of B; Wald=Wald statistics; df= degree of freedom; p=significance; Exp (B)=odds ratio.

CDSS= Calgary Depression Scale for Schizophrenia

GAF= Global Assessment of Functioning

PANSS= Positive and Negative Syndrome Scale

Omnibus Test of Model Coefficients: χ2=13.22, df=6, p=0.04

Hosmer and Lemeshow Test: χ2=8.83, df=7, p=0.265

**Figure1:**
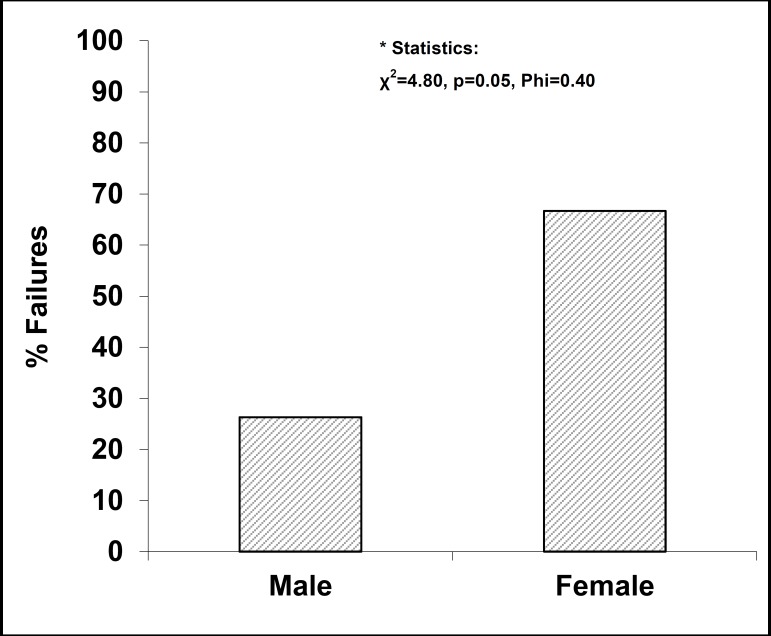
Gender Differences in Multiple Errands Test (MET) Performances

**Figure2:**
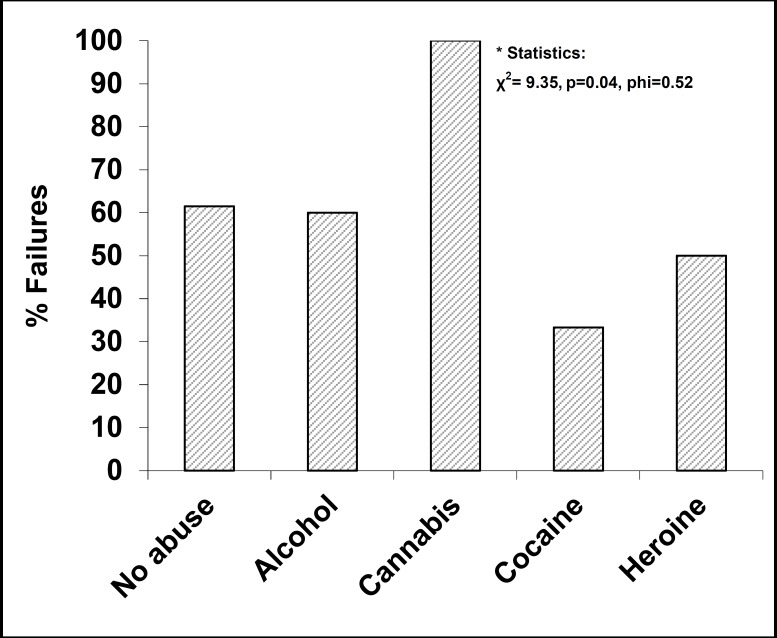
Substance Abuse and Differences in Verbal Fluency Performances

In addition, the model was overall significant (Omnibus test: χ2=13.22, df=6, p=0.04). An early age at onset was found to be associated with BACS Tower of London failure (OR=0.702, p=0.038). No further statistically significant associations were found (table 3). With regard to dichotomous variables, female schizophrenia patients showed more frequently failures in MET (total errors: χ2=4.80, p=0.05, Phi=0.40) (Figure 1) and in the HOTEL task (number of performed activities: χ2=5.25, p=0.04, Phi=0.42; number of correctly performed activities: χ2=5.25, p=0.04, Phi=0.42). In addition, cannabis abusers showed more frequently deficits on verbal fluency (χ2= 9.35, p=0.04, phi=0.52) (Figure 2) and executive functioning (Tower of London) (χ2= 11.67, p=0.02, phi=0.58) than alcohol/cocaine abusers. No further statistically significant associations were found.

## Discussion

The first result of our study is that clinically stabilized schizophrenia patients present impairment in several cognitive domains particularly in language comprehension (Token Test), social sensitivity (Faux Pas Test) and memory (Digit Symbol Coding). This finding supports the statement that schizophrenia cognitive dysfunction is not only associated to acute re-exacerbations, but it remains during the course of illness (55, 56). This is the reason why specific cognitive impairments have been recently proposed that neurocognitive markers of schizophrenic illness by international biological societies (57).

The main result of the present paper is that an early age at onset is associated with severity of frontal cognitive impairment (Tower of London). Frontal cognitive disability limits rehabilitation programs and impairs quality of life so that our findings confirm the view that early-onset schizophrenia patients have very poor prognosis (58). It is actually debated if cognitive dysfunction can be associated with brain changes that have been found in early-onset schizophrenia patients by recent neuroimaging researches (59-63). Interestingly, our data show that frontal dysfunctions do not result to be associated with age so that they probably emerge in first years of illness and keep stable over time. On the basis of this hypothesis, primary (identification of high-risk population) and secondary prevention programmes (early diagnosis) become preeminent to prevent or at least limit cognitive impairment and improve outcome of early-onset patients (64).

The second result is a worse social cognition in female schizophrenia patients respect to male ones. This finding is surprising as female gender is generally considered having a better outcome than schizophrenic males especially in case of a long duration of illness (65, 66). Perhaps these are specific cognitive deficits of female gender as in our knowledge this is the first study assessing social cognition in a sample of schizophrenia patients by Torralva’s neuropsychological battery. The results suggest that endophenotypes in schizophrenia may be sex-specific (67).

Finally, cannabis abuse appears to be associated with a more severe verbal/executive impairment in comparison with alcohol/cocaine abuse. It is traditionally stated that schizophrenic cannabis abusers show better neuropsychological performances than non-abusers in light of a baseline minor cognitive impairment (68). The discordant results of this paper can be explained by the long duration of illness of our sample: it can be hypothesized that cannabis-related cognitive impairment may be progressive during the course of schizophrenia, while cognitive impairment may be static in cocaine abusers (69).

## Limitation

Limits of the present study have to be shortly described. First the sample size is small, but this is partially due to the selection of patients in mono-therapies to limit the impact of medications on cognition. Second, possible confounding factors such as different antipsychotic mono-therapies might be biased the results, even though the available antipsychotics do not have a clear effect on cognition. Third gender imbalance in our sample might have influenced the study results. In contrast the naturalistic study design has the advantage to be more adherent with clinical practice. Studies with larger samples and possibly drug-naives could be useful to confirm the 

data of this article. 

## Conclusion

Stabilized schizophrenia patients show marked cognitive impairment especially regarding memory and social sensitivity. Early age at onset is associated with even a more compromised neuropsychological state with worse outcomes on executive functioning. Female gender appears to be associated with a poorer social cognitive functioning and cannabis abuse with impairment in verbal fluency and executive functioning.
